# Stochastic Simulation of Pattern Formation in Growing Tissue: A Multilevel Approach

**DOI:** 10.1007/s11538-018-0454-y

**Published:** 2018-06-20

**Authors:** Stefan Engblom

**Affiliations:** Division of Scientific Computing, Department of Information Technology, Uppsala University, 751 05 Uppsala, Sweden

**Keywords:** Reaction–diffusion master equation, Discrete Laplacian cell mechanics, Single-cell model, Cell population model, Notch signaling pathway, 60J28, 92-08, 65C40

## Abstract

We take up the challenge of designing realistic computational models of large interacting cell populations. The goal is essentially to bring Gillespie’s celebrated stochastic methodology to the level of an interacting population of cells. Specifically, we are interested in how the gold standard of single-cell computational modeling, here taken to be spatial stochastic reaction–diffusion models, may be efficiently coupled with a similar approach at the cell population level. Concretely, we target a recently proposed set of pathways for pattern formation involving Notch–Delta signaling mechanisms. These involve cell-to-cell communication as mediated both via direct membrane contact sites and via cellular protrusions. We explain how to simulate the process in growing tissue using a multilevel approach and we discuss implications for future development of the associated computational methods.

## Introduction

An important challenge in computational cell biology is to study the emergent behavior of single-cell pathways at the scale of a large interacting cell population. In this paper, we tackle this challenge by, in essence, attempting to generalize Gillespie’s stochastic simulation methodology to the level of the multicellular environment. In order to do so, clearly, the modeling physics of the extracellular space, of the cell population, and of the single cells need to be prescribed. A suitable computational methodology should additionally allow for cell-to-cell signaling in a flexible and general way. There are several possible interesting applications for such a kind of modeling framework, regulating processes in embryonic development, angiogenesis, neurogenesis, wound healing, and tumor growth, to mention just a few.

In the interest of focusing our work around a concrete, yet fairly demanding modeling situation, we pick as our target a specific set of network models which involve single-cell pathways together with non-trivial signaling between the individual cells. The Notch signaling pathway is a highly conserved mechanism which is present in most multicellular organisms (Artavanis-Tsakonas et al. 1999), ranging from, e.g., *Drosophila* and *C. elegans* to mammals. Indeed, the fundamental importance of Notch signaling made it an early target for mathematical models (Collier et al. 1996), where feedback regulation between neighbor cells was modeled. It has since been realized that cell-to-cell signaling not only is short range, taking place at direct junctional contact sites, but also is mediated via long range cellular protrusions (Cohen et al. 2010). Mathematical models including these effects have recently been investigated (Sprinzak et al. 2011; Hadjivasiliou et al. 2016), and we choose a family of such models as the concrete target in this paper.

To be able to realistically resolve the geometrical details of the single cell, unstructured meshes (e.g., triangularizations) stand out as a ubiquitous tool. Also, an important part of Dan Gillespie’s heritage to computational biology is that noisy cellular processes at the molecular level should be understood in a *stochastic* framework. These observations together suggest the *reaction–diffusion master equation* (RDME) over an unstructured mesh (Engblom et al. 2009), and we shall regard it herein as a gold standard in single-cell modeling. The RDME is based on first principles and is reasonably effective computationally. Additionally, this description, or simplified versions of it, has been successful at delivering important insights for a range of cellular phenomena (Fange and Elf 2006; Raj and van Oudenaarden 2008; Lestas et al. 2010; Barkai and Leibler 2000).

At the scale of a population of cells, cell-based computational modeling is an *in silico* approach to test hypotheses concerning the contributions of various mechanisms to observed macro-level behaviors. Examples of recent applications of cell-based models include embryonic development (Atwell et al. 2015), wound healing (Vermolen and Gefen 2013; Ziraldo et al. 2013), and tumor growth (Naumov et al. 2011). The natural analog of the RDME at the cell population level is found in the class of *on-lattice* cell-based models. As in the RDME, space is here discretized in a grid of voxels over which the cells are distributed. State update rules are then formulated over this grid where signaling processes and factor concentrations may be included via, e.g., differential equations (Robertson-Tessi et al. 2015).

In this work, we will focus on the novel on-lattice method proposed in Engblom et al. (2018), which is promising from a scaling point of view, yet also is very expressive. The method is referred to as *discrete Laplacian cell mechanics* (DLCM) and is formed by developing constitutive equations for the dynamics of the cell population at a given discretization of space. The update rules are stochastic and are established from global calculations. Importantly, the simulations take place in continuous time, thus allowing for a meaningful coupling to arbitrary continuous-time processes, including inter-cellular signals. In summary, we focus our attention to single-cell models described in the RDME framework and the main contribution of the paper is to investigate the feasibility of the two-level RDME-DLCM approach.

In the next section, we work through the specific, but fairly general, pattern forming mechanism we wish to study and we subsequently express it within the RDME-DLCM computational framework. As will be demonstrated, this enables us to simulate a range of intriguing patterns in an unprecedented detailed and bottom-up fashion. The paper is concluded with a discussion of some ideas concerning possible future developments of the presented computational methodology.

## Models and Methods

Below we start by presenting the specific Notch pathway model, we will target in the paper. Throughout we consider a single non-dimensional model, originating from an attempt to map to the situation of explaining the organization of bristles on the *Drosphila* notum (Cohen et al. 2010). Such patterns are remarkably precise and are therefore good model systems to study the genetic basis of pattern formation. The model we decided to employ can be found in Hadjivasiliou et al. (2016). We make a slight extension of the model in Sect. 2.2 by bringing it into the spatial setting, essentially by deciding on a system volume and settling for suitable diffusion constants. In Sect. 2.3, we explain how to use the methodology in Engblom et al. (2018) to efficiently simulate a growing cell population. The two computational layers, i.e., the single-cell and the cell population layer, are put together in Sect. 2.4 where we present a few selected simulation results. In order to concentrate on the possibilities with the computational framework, we select spatial and cell population parameters rather freely, and we do not claim our resulting model to map to any specific real-world scenario.

### Protrusion Mediated Notch–Delta Pattern Formation

An early attempt to mathematically explain pattern formation mechanisms in tissue without resorting to the postulated existence of *morphogens*, i.e., as done early on by Turing (1952), was based on lateral inhibition with feedback (Collier et al. 1996). This mechanism takes place in between the transmembrane proteins Notch and Delta, respectively. In a non-dimensional setting, with $(n_i,d_i)$ denoting the Notch- and Delta concentrations within cell *i*, the original model has the form (Collier et al. 1996)

1 $$\begin{aligned}&\left. \begin{array}{rcl} n'_i &{}=&{} f(\langle d \rangle _i)-n_i \\ d'_i &{}=&{} \text{ const. } \times \left( g(n_i)-d_i \right) \\ \end{array} \right\} \end{aligned}$$

where $'$ denotes differentiation with respect to time and where $\langle d \rangle _i$ denotes the incoming Delta signal, averaged from the cells surrounding cell *i*. In (1), *f* and *g* denote monotonically increasing and decreasing functions of their single argument, respectively.

Whereas the classical Notch–Delta model gives an alternating pattern of ’black’ (e.g., high Delta) and ’white’ (low Delta), patterns in Nature are often much more involved, e.g., with sparse dots, or spots, stripes, and labyrinth-like patterns. In an attempt to explain the dot-like pattern of the notum (dorsal portion of the thoracic segment) of *Drosophila*, communication of Delta via cellular protrusions was added to the model in Cohen et al. (2010). Later details were added in Sprinzak et al. (2011), Hadjivasiliou et al. (2016), including differential weighting of the incoming signals and an addition of the concentration of a Notch reporter molecule $r_i$. More specifically, the model from Hadjivasiliou et al. (2016) reads

2 $$\begin{aligned}&\left. \begin{array}{rcl} n'_i &{}=&{} \beta _n-\frac{\langle d_\mathrm{in} \rangle n_i}{k_t}-\frac{d_in_i}{k_c}-n_i \\ d'_i &{}=&{} \beta _d\frac{1}{1+r_i^m}-\frac{d_i\langle n_\mathrm{in} \rangle }{k_t}- \frac{d_in_i}{k_c}-d_i \\ r'_i &{}=&{} \beta _r\frac{(\langle d_\mathrm{out} \rangle n_i)^s}{k_{rs}+(\langle d_\mathrm{out} \rangle n_i)^s}-r_i \\ \end{array} \right\} \end{aligned}$$

In (2), Delta $d_i$ is down-regulated by the Notch reporter $r_i$, which in turn is up-regulated by Notch $n_i$ and, respectively, the outgoing Delta $\langle d_\mathrm{out} \rangle$, as discussed below. The Hill coefficients in these regulations are taken to be $m = s = 2$ throughout the paper.

**Fig. 1:**
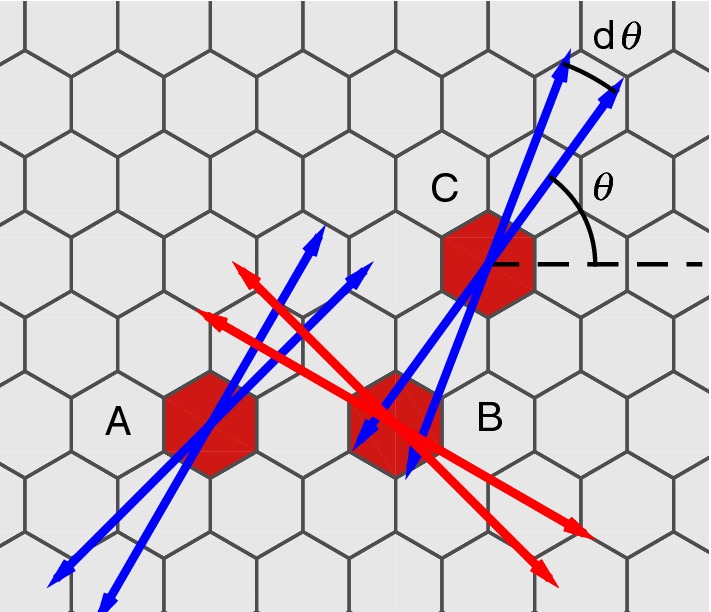
Signaling via protrusions: the symmetric contact $A \longleftrightarrow B$ is protrusion mediated, $C \longrightarrow B$ is protrusion to membrane, and $B \longrightarrow C$ is membrane to protrusion. A junctional contact is also possible between neighbor cells (not shown). In the running model of the paper, the first two types of contacts are understood to be protrusional [superscript (*b*)], while the two latter types are junctional [superscript (*a*)]. Protrusions are parameterized by the protrusion length *l*, direction $\theta$, and angular width $d\theta$

The existence of protrusional communication clearly implies more cell-to-cell signaling possibilities (Fig. 1). In (2), the amount of incoming and outgoing Delta and Notch, respectively, is given by

3 $$\begin{aligned}&\left. \begin{array}{rcl} \langle d_\mathrm{in} \rangle &{}=&{} w_a \langle d \rangle _i^{(a)}+w_b \langle d \rangle _i^{(b)} \\ \langle d_\mathrm{out} \rangle &{}=&{} q_a \langle d \rangle _i^{(a)}+q_b \langle d \rangle _i^{(b)} \\ \langle n_\mathrm{in} \rangle &{}=&{} w_a \langle n \rangle _i^{(a)}+w_b \langle n \rangle _i^{(b)} \end{array} \right\} \end{aligned}$$

The superscript (*a*) and (*b*) denote the sum of the signal over all cells making *junctional* and, respectively, *protrusional* contact (see Fig. 1). More concretely,

4 $$\begin{aligned} \langle d \rangle _i^{(a)}&:= \sum _{j \in J(i)} d_j, \qquad \langle d \rangle _i^{(b)} := \sum _{j \in P(i)} d_j, \end{aligned}$$

in which *J*(*i*) and *P*(*i*) denote the set of junctional and protrusional contacts for cell *i*. A specific novelty with this model is $\langle d_{rm out} \rangle$, the total amount of bound Delta that leads to activation of the Notch receptor. Differential weighting of the signals is achieved by assuming different constant weights $[w_a,w_b,q_a,q_b]$ of the incoming signals.

To formulate a stochastic well-stirred interpretation of (2), we understand the concentrations (*n*, *d*, *r*) in cell *i* as absolute molecular counts (*N*, *D*, *R*) at some fix system volume $\Omega$. From (2), we propose the transitions

5 $$\begin{aligned}&\left. \begin{array}{lll} \emptyset \xrightarrow {\beta _n \Omega } N &{} \quad \emptyset \xrightarrow {\beta _d \Omega \, r_1} D &{} \quad \emptyset \xrightarrow {\beta _r \Omega \, r_2} R \\ N \xrightarrow {\langle D_\mathrm{in} \rangle /(k_t\Omega )} \emptyset &{} \quad D \xrightarrow {\langle N_\mathrm{in} \rangle /(k_t\Omega )} \emptyset &{} \quad N+D \xrightarrow {1/(k_c \Omega )} \emptyset \\ N \xrightarrow {1} \emptyset &{} \quad D \xrightarrow {1} \emptyset &{} \quad R \xrightarrow {1} \emptyset \end{array} \right\} \end{aligned}$$

in terms of

6 $$\begin{aligned} r_1&\equiv \frac{1}{1+(R/\Omega )^2}, \quad r_2 \equiv \frac{(\langle D_\mathrm{out} \rangle N/\Omega ^2)^2}{k_{rs}+(\langle D_\mathrm{out} \rangle N/\Omega ^2)^2}. \end{aligned}$$

**Fig. 2:**
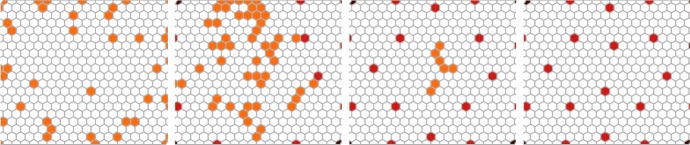
Stochastic pattern development over a static population of cells. Here the 0-dimensional interpretation of model (5) with system volume $\Omega = 400$ is employed and is simulated using Gillespie’s direct method. From left to right: time $t = [4,20,40,200]$. Color codes low (white) and high Delta (orange, light, and dark brown). Parameters are adopted from Hadjivasiliou et al. (2016): $[\beta _n,\beta _d,\beta _r] = [100,500,3 \cdot 10^5]$, $[k_t,k_c,k_{rs}] = [2,0.5,10^7]$, protrusion length $= 3.5$ cell radii, angular width $= 2\pi$, $[w_a,q_a,w_b,q_b] = [1,1,1,1]$

We test this model over a static hexagonal grid using Gillespie’s direct method for the simulation (Fig. 2). To practically evaluate the various incoming signals $\langle D_\mathrm{in} \rangle$, $\langle N_\mathrm{in} \rangle$, and $\langle D_\mathrm{out} \rangle$, we settle for a small time-step $d\tau$ and make the approximation that signals (3) remain constant in the interval of time $[t,t+d\tau ]$, in line with, e.g., the approach taken in Puchalka and Kierzek (2004). The time interval was here chosen in a quite conservative way such that a forward Euler step of original ODE model (2) would imply a 5% change of state in a norm-wise sense,

7 $$\begin{aligned} d\tau&= 0.05 \times \frac{\Vert x\Vert }{\Vert f(x)\Vert }, \end{aligned}$$

where $x = [n,d,r] {= [N,D,R]/\Omega }$ is the concentration vector for the whole cell population and where $f(\cdot )$ is the right-hand side of (2).

Simulation results for this model when starting from a random initial configuration are summarized in Fig. 2 at a system volume $\Omega = 400$. We now proceed to make an immediate spatial extension of this model.

### Spatial Stochastic Reaction–Diffusion Models of Single Cells

Living cells are inherently inhomogeneous objects, and the assumption of well-stirredness can rightly be questioned (Dobrzyński et al. 2007; Fange and Elf 2006). The reaction–diffusion master equation (RDME) attempts to strike a balance between accuracy and computational efficiency (Gardiner 2004). Here the domain under consideration is discretized in small enough compartments, or *voxels*, such that diffusion is enough to regard each voxel as well stirred. Diffusion in between voxels is handled as a special set of reactions with rates obtained so as to match with macroscopic diffusion properties (Engblom et al. 2009). An efficient algorithm for spatial stochastic simulation is the Next subvolume method (NSM) (Fange and Elf 2006), which can be thought of as a blend of Gillespie’s Direct method with the Next reaction method. The algorithm is summarized in “Appendix A.”

We like to regard the RDME as a kind of “gold standard” in single cell modeling. Although it is possible to make more accurate computational models in the sense of bringing in more physical details, say at the level of single molecules (Andrews and Bray 2004), this comes at large computational costs. There is also the issue with uncertainties in rate parameters, and the risk of over-modeling in many situations of practical biological interest.

**Fig. 3:**
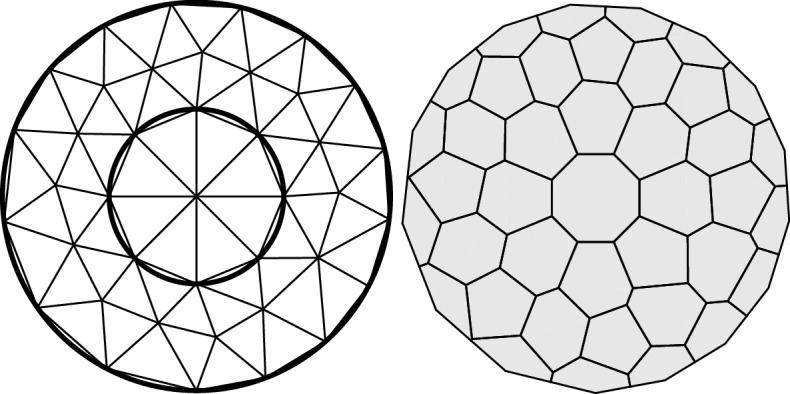
Single-cell discretization mesh used in the running model. Left: triangularization of a basic two-dimensional cell geometry consisting of a cytoplasm and a nuclei, right: the associated dual mesh consisting of the computational compartments (voxels)

To illustrate the single cell population-level approach we have in mind, we make an immediate version of pathway model (5) as follows. As a single-cell discretization, we take the triangularization depicted in Fig. 3 which consists of a modest number of 40 voxels. We make no particular distinction of the membrane, the cytoplasm, or the nuclei, but allow all reactions in (5) to take place in all of the voxels. We let the geometry be of total volume $\Omega = 400$ and use the scalar diffusion constant $1/\Omega$ across the whole cell geometry and for all species [*N*, *D*, *R*]. Although there are clearly many potential improvements to this basic model, it will serve as an interesting load case to our simulation approach. We thus have to postpone for another occasion the interesting quest for additional modeling realism including, e.g., nuclei- and membrane specific transitions.

### Stochastic Simulation of Growing Cell Populations

Given the relative efficiency of the RDME approach, one can wonder if not a similar idea could be useful at the cell population level. Unlike the various molecules inside the living cell, however, cells in multicellular structures do not generally diffuse around freely. Instead, cells may actively crawl, adhere to other cells, and are pushed into position. An RDME-like framework for this situation was recently developed, and we now briefly review this idea (Engblom et al. 2018).

**Fig. 4:**
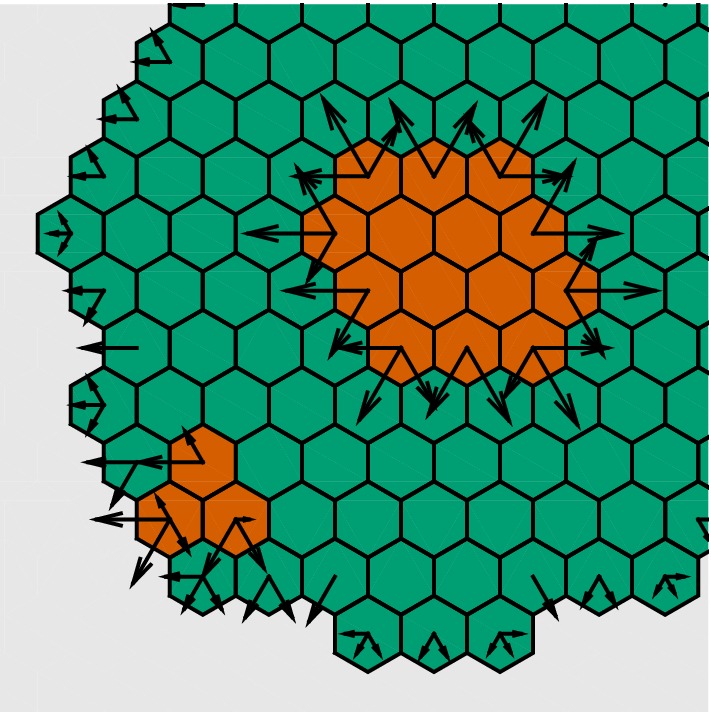
Schematic illustration of the DLCM method (adapted from Engblom et al. 2018). Green voxels contain single cells and red voxels contain two cells, giving rise to a cellular pressure. A discrete Laplace operator is employed to propagate this pressure, thus inducing a rate to move for the cells in the voxels as indicated by the arrows. Cells in boundary voxels may move into empty voxels and cells in doubly populated voxels may move into voxels containing fewer cells

We assume a two- or three-dimensional computational grid consisting of voxels $(v_i)$, $i = 1,\ldots ,N_{{\mathrm{vox}}}$ (Fig. 4). At this level of description, the individual cells are placed in the single voxels of a typically structured grid, e.g., squares or hexagonals, although unstructured grids are certainly also a possibility. As in the RDME, it is fundamental that a consistent Laplace operator may be defined over this grid, hence the name discrete Laplacian cell mechanics, or DLCM.

The voxels are either empty or may contain a certain number of cells, here taken to be either 1 or 2. If the number of cells in all voxels is either 0 or 1, i.e., below the carrying capacity, then the system is in equilibrium given that there are no other active processes. If the number of cells in one or more voxels is 2, a cellular pressure is exerted toward the neighbor voxels. This state is eventually changed by an event where one of the cells moves into a neighboring voxel, and then, the pressure distribution changes. This process continues until, possibly, the system relaxes into equilibrium.

**Fig. 5:**
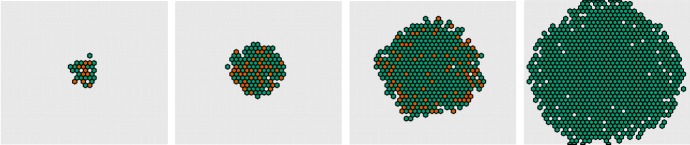
Growth of cell population. Nutrition is made available at the boundaries and diffuses through the population and is consumed by individual cells. From left to right: time $t = [14.4,21.8,31.9,197.9]$

What is then the physics for this cellular pressure which drives the shape of the cell population? A detailed derivation is made in Engblom et al. (2018), but in short, the answer is that a suitable physics is formed by letting the pressure be spread according to the negative Laplacian, and with source terms for all over-occupied voxels (see Fig. 4). Let $(u_i)$, $i = 1,\ldots ,N_{{\mathrm{vox}}}$ denote the number of cells in voxel *i* and let $(p_i)$ be the corresponding cellular pressure. Denote by $\Omega _h$ the subset of voxels $v_i$ for which $u_i \not = 0$ and let $\partial \Omega _h$ denote the discrete boundary, the set of unpopulated voxels that share an edge with a voxel in $\Omega _h$. At any instant in time *t*, we solve for the cellular pressure,

8 $$\begin{aligned} -L p&= s(u), \quad i \in \Omega _h, \end{aligned}$$

9 $$\begin{aligned} p_i&= 0, \quad i \in \partial \Omega _h, \end{aligned}$$

in which *L* is a consistent discretization of $\Delta$ over $\Omega _h$ and where the source term is $s(u_i) = 0$ for $u_i \le 1$ and $s(u_i) = 1$ whenever $u_i = 2$. This normalization ensures that $p = 0$ at equilibrium. It is doable to rely on this setup also for unstructured meshes by postulating that the cellular pressure is proportional to the difference in volume occupancy and voxel volume. However, there are biological specifics which should rightly be considered in this case, such as adhesion effects in voxels populated under their carrying capacity, and also details concerning the volume characteristics of the individual cells.

The movements in the cell population are induced by a pressure gradient between two voxels. Denote by $I(i \rightarrow j) = I_{ij}$ the current from voxel $v_i$ to the neighbor voxel $v_j$. This current is found by integrating the pressure gradient across the edge between the two voxels,

10 $$\begin{aligned} I_{ij}&= -\int _{v_i \cap v_j} \nabla p(x) \, \cdot \mathrm{d}S = \frac{e_{ij}}{d_{ij}} (p_i-p_j), \end{aligned}$$

with $d_{ij}$ the distance between voxel centers and $e_{ij}$ the common edge length. The *rate* for the event that one cell moves from voxel *i* to *j* is taken to be

11 $$\begin{aligned} R(i \rightarrow j) = R_{ij} = D I_{ij}, \end{aligned}$$

where the conversion factor *D* may depend on position and on the type of movement. We take $D = 0$ for movements into voxels containing an equal number of cells, thus limiting the cellular movements to less crowded voxels only.

The implied simulation method is event based and is directly based on Gillespie’s direct method; see “Appendix A,” Algorithm A.2. Chiefly, for any given state of the cell population, the rates of all events are determined and the time and kind of the next event is sampled. Until the time of this next event, any other processes local to each voxel may be simulated. When the event is processed, a new cell population state is obtained and the process starts anew.

**Fig. 6:**
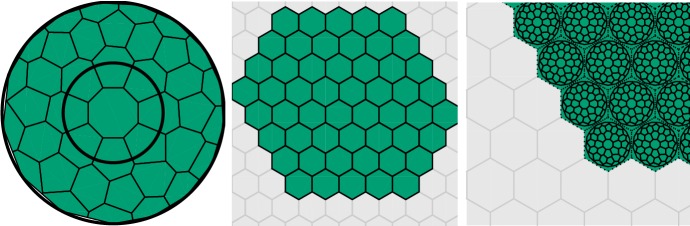
Inner-outer structure of the RDME-DLCM simulation approach. Left: the single-cell model is simulated across all cells in the population displayed in the middle. The coupling between the cells which is required to capture, e.g., signaling processes, can be handled by a split-step time-discretization strategy. Middle: this continues until an event at the population layer is sampled. After executing this event and updating the internal states of the individual cells accordingly, the single-cell model is evolved anew. Right: the effective grid induced by this computational process can be understood as replicas of the single-cell discretization

We exemplify the process by growing a small population of 1000 cells, starting from a single cell and allowing it to proliferate at a certain rate provided it has enough concentration of “nutrition.” The nutrition is distributed at the boundaries $\Omega _h$ of the cell population, and we let it diffuse by the Laplace operator. At any given time, cells consume nutrition for their own metabolism and so this scheme will favor the proliferation of cells near the boundary where the nutrition concentration is the highest (see Fig. 5). In the next section, we proceed by coupling this DLCM layer growth process to the previously developed RDME-layer description of the Notch–Delta pattern formation mechanism. Hence, the fine RDME discretization as depicted in Fig. 3 is used to describe the physics of the individual cell, whereas the DLCM grid is used for the cell population.

### A Range of Notch–Delta Patterns in Growing Tissue

In the present case, the cellular growth process is independent from the single-cell model and the two layers can be conveniently simulated under a simple one-way coupling. That is, in a first run we simulate the growth process and record all associated events separately. Next, the RDME-layer model is simulated in continuous time and in between all the recorded events, thus realizing the overall dynamics. This coupling restriction is only used for convenience here: the DLCM simulation, cf. Algorithm A.2 in “Appendix A,” allows for a completely general two-way coupling. However, in practice, it usually makes sense to assume some kind of scale separation between the two layers (Shimoni et al. 2011), such that the inner split-step $d\tau$ need not be much smaller than what is required to resolve the dynamics at the outer layer. Regardless of such an assumption, the split-step strategy can be expected to be strongly convergent in the stochastic sense (Engblom 2015), although the split-step $d\tau$ might be severely restricted for accuracy reasons.
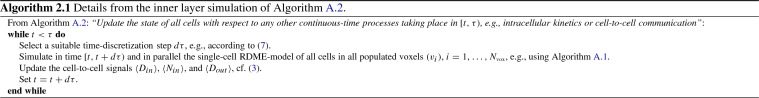


In Algorithm 2.1, we expand the details of the inner layer simulation where the discretization in time chunks $[t,t+d\tau )$ is made explicit. It follows that the assumption made here is essentially that the Notch–Delta dynamics takes place on a faster timescale than the growth process, a quite reasonable assumption in this case. Without this assumption, the simulation efficiency will deteriorate whenever $d\tau$ for accuracy reasons has to be chosen small.

**Fig. 7:**
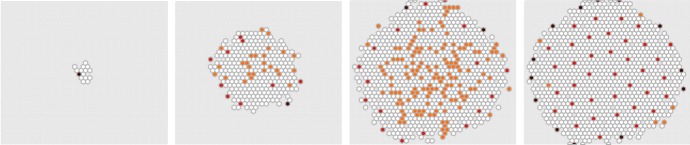
Notch–Delta-Reporter model in a growing domain. The RDME is used to describe the individual cells and the DLCM models the cell population growth. The parameters are as in Figs. 2 and 5

**Fig. 8:**
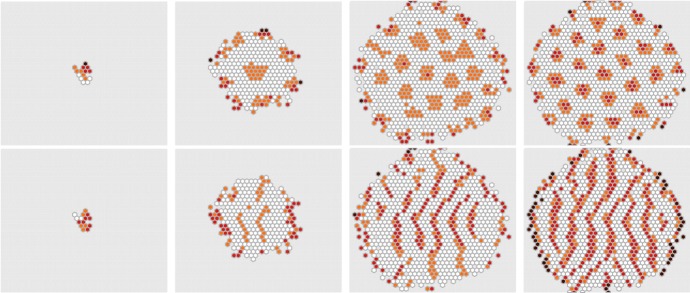
Top row: development of spots by differential weighting. The parameters are as in Fig. 7, but with $[w_a,q_a,w_b,q_b] = [1,0.001,0.06,0.06]$. Bottom row: the effect of polarized protrusions. Here the protrusions stretch horizontally, $\theta = \pm \pi$, at an angular width $[0,\pi /20]$ and a length of 5 cell radii. The parameters are again as in Fig. 7 but with $[w_a,q_a,w_b,q_b] = [1,0.001,0.2,0.15]$

The coupled inner-outer algorithm can be understood as a highly detailed simulation on a very fine mesh covering the whole cell population (see Fig. 6). In Fig. 7, we visualize results from the full Notch–Delta-Reporter model (2), interpreted in a spatial stochastic sense as explained in Sect. 2.2, and simulated together with the growth process as described in Sect. 2.3. A small extension to the model was made here in that proliferating cells randomly share Notch and Delta in between each other, thus adding some noise to the overall dynamics. The model itself exhibits a range of intriguing patterns as discussed in Hadjivasiliou et al. (2016). Two such examples are further investigated in Fig. 8.

## Conclusions

The main focus of this paper has been to investigate the feasibility of a two-level RDME-DLCM approach. We choose the RDME description of a single cell as a gold standard modeling approach. This is a detailed, flexible, yet also comparably effective simulation methodology. At the cell population level, the related DLCM method was used and the two layers of description were coupled together with relative ease.

The main reason this combination is convenient is the fact that both layers take place in continuous time and can be simulated by Gillespie-style event-driven algorithms. We also point out that the overall method combination is promising from the point of view of deriving approximate simulation algorithms, as, for example, shown in detail for the RDME framework in Chevallier and Engblom (2018). A concrete example is that, for practical reasons in the implementation, we had to discretize time for the cell-to-cell signaling process of the model, cf. (7). Although not discussed here, one can expect that this method has a strong error of order $O(d\tau ^{1/2})$ Engblom (2015). Since we selected a quite conservative time step in (7), we believe that our implementation is a bit inefficient in that the time-step restriction is too restrictive given the accuracy demands of the application at hand. This is an issue which could clearly be of interest to target in future research toward faster algorithms. Other related ideas are deterministic-stochastic hybrid algorithms Chevallier and Engblom (2018), Haseltine and Rawlings (2002), Lo et al. (2016) and, more generally, multiscale solvers based on ideas from stochastic homogenization techniques (Ea et al. 2005; Cao et al. 2005b, a; Cao and Petzold 2008). At the DLCM layer, the computational bottleneck lies in factorizing the Laplacian operator. Real savings in computing time here can be expected from employing traditional multigrid techniques Stüben (2001), Trotter et al. (2001).

Lastly but not the least, the computational framework described clearly opens up for many interesting applications where the emerging cell population behavior of detailed whole-cell models is to be approached.

### Availability and Reproducibility

The computational results can be reproduced within the upcoming release 1.4 of the URDME open-source simulation framework Drawert et al. (2012), available for download at www.urdme.org.
